# Right Atrial Clot Formation Early after Percutaneous Mitral Balloon Valvuloplasty

**DOI:** 10.1155/2016/3058015

**Published:** 2016-12-25

**Authors:** Ahmet Hakan Ateş, Uğur Arslan, Aytekin Aksakal, Huriye Yücel, İlksen Atasoy Günaydın, Adem Ekbul, Mehmet Yaman

**Affiliations:** Department of Cardiology, Health Sciences University Samsun Training and Research Hospital, Samsun, Turkey

## Abstract

Mitral balloon valvuloplasty which has been used for the treatment of rheumatic mitral stenosis (MS) for several decades can cause serious complications. Herein, we presented right atrial clot formation early after percutaneous mitral balloon valvuloplasty which was treated successfully with unfractioned heparin infusion.

## 1. Introduction

Rheumatic mitral valve stenosis is a chronic manifestation of rheumatic carditis which is seen especially in developing and undeveloped countries. Although all of the cardiac valves may be involved by rheumatic carditis, mitral valve is the most commonly affected one and stenosis of this valve occurs as a result of leaflet thickening, commissural fusion, and chordal shortening. Before technical development of transcatheter interventions, most patients with symptomatic mitral stenosis were treated with surgical mitral commissurotomy, either open or closed [1]. After the development of percutaneous mitral balloon valvuloplasty (PMBV) technique, it has become the first choice for the treatment of patients with mitral stenosis who have Wilkins score < 8. However this procedure has several life threatening complications like hemopericardium, cardiac tamponade, clot formation, and cardiac rupture [2, 3].

## 2. Case Report

**Figure 1:**
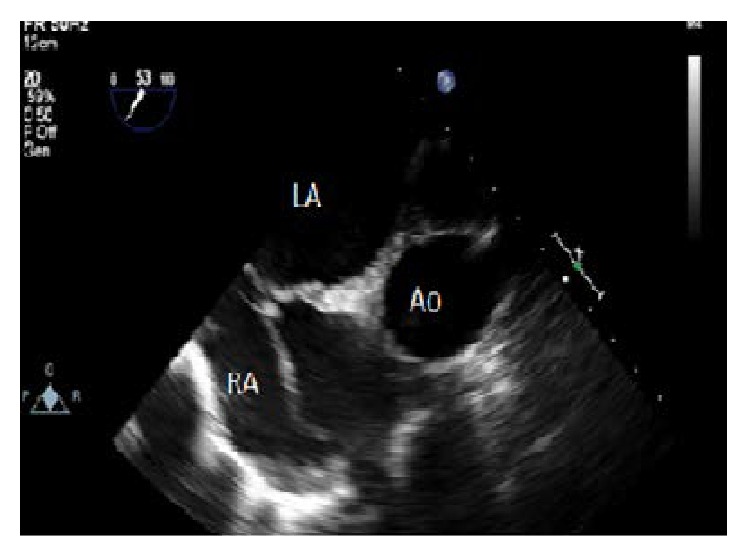
Thrombus formation in right atrium after the procedure, transesophageal echocardiography image of thrombus (Ao: Aorta, LA: Left Atrium, and RA: Right Atrium).

A 40-year-old female was admitted to our department with palpitation and exertional dyspnea with an increasing intensity in the last year. She had no medical history before and her ECG was in sinus rhythm with a rate of 77 beats/min. In her physical examination, we found 2/4 middiastolic murmur at the apex and mitral opening snap. Transthoracic echocardiography was performed which exposed rheumatic mitral stenosis with a valve area of 1.1 cm^2^ and Wilkins score was calculated to be 7. The patient also had mild mitral regurgitation (MR) with a systolic pulmonary artery pressure (PAP) of 55 mmHg. We decided to perform PMBV for mitral stenosis because of increased clinical symptoms in the last year and increased PAP. Before operation, we performed transesophageal echocardiography (TEE) to evaluate the structure of mitral valve (leaflets, subvalvular apparatus, papillary muscles, and chords) and to exclude thrombus formation in left atrial appendage. No thrombus was found in left atrium and the mitral valve was found to be suitable for PMBV without any severe valvular calcification or significant chordal fusion. There was only mild MR in TEE. The patient underwent PMBV thereafter. Septal puncture was performed with Brockenbrough needle under fluoroscopic guidance through femoral vein approach with TEE support. Just after transseptal puncture 7500 units unfractionated heparin was administered and we performed PMBV using Inoue Balloon catheter (Toray, Tokyo, Japan) system with a 26 mm balloon. Just after this, we checked mitral valve area and function with transthoracic echocardiography. The MVA was calculated to be 1.6 cm^2^ without a significant increase in mitral regurgitation. Then, we performed second valvuloplasty procedure successfully with a 28-mm balloon. The procedure was performed successfully with a MVA of 1.9 cm^2^. However, a highly mobile fresh thrombus was observed in the right atrium moving freely at a size of 9 × 31 mm (Figure 1). Afterwards, the patient was managed with continuous unfractionated heparin immediately targeting the partial thromboplastin time between 50 and 70 seconds. After 24-hour heparin infusion, transthoracic and transesophageal echocardiography were performed again without any clot formation. No sign of pulmonary or systemic embolism was found after the disappearance of the right atrial thrombus.

## 3. Discussion

Percutaneous mitral balloon valvuloplasty is a safe and effective procedure with a high success rate for the treatment of rheumatic mitral stenosis. This procedure has also several life threatening complications such as acute pulmonary edema, myocardial infarction, cardiac perforation, cardiac tamponade, acute mitral regurgitation, hemopericardium, thrombus formation, systemic and pulmonary embolism, permanent atrial septal defect, cardiogenic shock, and neurological deficit with an overall incidence of 12% [1, 2]. Most of these severe complications occur especially during transseptal puncture [3].

Herein, we reported a patient who developed right atrial thrombus formation during the PMBV procedure. There have been very few case reports about right atrial thrombus formation during or after PMBV in literature. In 2013, Salehi et al. reported a right atrial clot formation during PMBV which was treated successfully with surgery [4]. To the best of our knowledge, this is the first case of right atrial clot formation that occurred after PMBV which disappeared as early as 24 hours after unfractioned heparin infusion. In another case in which the right atrial clot formation after PMBV was treated with heparin, thrombus disappeared 72 hours after heparin infusion [5]. The exact mechanism of right atrial thrombus formation is unknown. But damage to the endocardial surface of right atrium during transseptal puncture can play a key role for thrombus formation. Another possible reason is that low inner pressure and slow blood flow in the right atrium when combined with the presence of foreign catheters trigger a prothrombotic state within this heart chamber [6]. In our case, heparin was administered just after transseptal puncture and in the literature there are some cases related to thrombus formation after the termination of procedure despite heparin administration right after transseptal puncture [7]. Until heparin administration, the right side of heart was prone to thrombus formation, which might explain the clot formation in our patient.
